# Cannabidiol Affects MK-801-Induced Changes in the PPI Learned Response of Capuchin Monkeys (*Sapajus* spp.)

**DOI:** 10.3389/fphar.2017.00093

**Published:** 2017-02-27

**Authors:** Patricia G. Saletti, Rafael S. Maior, Marilia Barros, Hisao Nishijo, Carlos Tomaz

**Affiliations:** ^1^Primate Center and Laboratory of Neurosciences and Behavior, Department of Physiological Sciences, Institute of Biology, University of BrasiliaBrasilia, Brazil; ^2^Department of Clinical Neuroscience, Psychiatry Section, Karolinska Institutet, Karolinska University HospitalStockholm, Sweden; ^3^Department of Pharmaceutical Sciences, School of Health Sciences, University of BrasiliaBrasilia, Brazil; ^4^System Emotional Science, Graduate School of Medicine and Pharmaceutical Sciences, University of ToyamaToyama, Japan; ^5^Neuroscience Research Group, University CEUMASão Luís, Brazil

**Keywords:** cannabidiol (CBD), MK-801, monkey, prepulse inhibition, learning, memory

## Abstract

There are several lines of evidence indicating a possible therapeutic action of cannabidiol (CBD) in schizophrenia treatment. Studies with rodents have demonstrated that CBD reverses MK-801 effects in prepulse inhibition (PPI) disruption, which may indicate that CBD acts by improving sensorimotor gating deficits. In the present study, we investigated the effects of CBD on a PPI learned response of capuchin monkeys (*Sapajus* spp.). A total of seven monkeys were employed in this study. In Experiment 1, we evaluated the CBD (doses of 15, 30, 60 mg/kg, i.p.) effects on PPI. In Experiment 2, the effects of sub-chronic MK-801 (0.02 mg/kg, i.m.) on PPI were challenged by a CBD pre-treatment. No changes in PPI response were observed after CBD-alone administration. However, MK-801 increased the PPI response of our animals. CBD pre-treatment blocked the PPI increase induced by MK-801. Our findings suggest that CBD’s reversal of the MK-801 effects on PPI is unlikely to stem from a direct involvement on sensorimotor mechanisms, but may possibly reflect its anxiolytic properties.

## Introduction

Cannabidiol (CBD) is one of the several compounds extracted from the Cannabis plant reported to have different therapeutic applications (Baker et al., 2003). These seem to include general systemic anti-inflammatory (Mechoulam et al., 2002; Vilela et al., 2015) and antioxidant properties (Hampson et al., 1998), as well as more specific effects in different neurological disorders, such as epilepsy (Mechoulam et al., 2002), anxiety (Zuardi et al., 1982; Campos and Guimarães, 2008), and schizophrenia (Zuardi et al., 1991; Leweke et al., 2012).

The antipsychotic effects, in particular, may result from the antagonistic action of CBD on cannabinoid type 1 (CB1) and 2 receptors (CB2; Thomas et al., 2007). Zuardi et al. (2012) suggested, however, that an interaction between CBD and anandamide is essential for the psychoactive effect, considering that CBD also increases the availability of this endocannabinoid via a reuptake inhibition mechanism (Mechoulam et al., 2002). The antipsychotic properties of CBD have also been linked to changes in glutamate signaling that results from the activation of the vanilloid TRPV type 1 receptor system. Anandamide (Di Marzo et al., 2001) and CBD (Cristino et al., 2006) are both known TRPV1 receptor ligands, and the pre-synaptic activation of this system increases the release of glutamate in psychosis-related areas of the brain (Xing and Li, 2007). Furthermore, CBD is reported to reverse the prepulse inhibition (PPI) disruption induced by the non-competitive glutamate NMDA receptor antagonist dizocilpine (MK-801) in murine models (Long et al., 2006; Gomes et al., 2014; Levin et al., 2014; however, see Gururajan et al., 2011), with this effect being antagonized by the TRPV1 receptor antagonist capsazepine (Long et al., 2006).

In fact, MK-801 has become a frequently used pharmacological tool to induce schizophrenic-like symptoms in pre-clinical experimental setups (Arai et al., 2008; Park et al., 2014; Saletti et al., 2015). Schizophrenic patients commonly demonstrate significant deficits in sensorimotor gating (Braff et al., 2001). This dissociative anesthetic is also reported to disrupt the PPI responding of rodents (Hoffman et al., 1993), as well as to induce cognitive (Hikichi et al., 2013; Karamihalev et al., 2014) and social recognition deficits (Yoshimi et al., 2015) and hyperlocomotion (Park et al., 2014; Basurto et al., 2015). However, to our knowledge, the effects of CBD on the PPI response of non-human primates (NHP) have yet to be assessed. It is known that manipulations of learning and memory-related areas, such as the hippocampus, can alter PPI responding (Kohl et al., 2013). Compared to rodents, NHPs have a distinct motor response to cannabinoid-related substances (Meschler et al., 2001) and display higher CB1 receptor densities in memory-related areas (Ong and Mackie, 1999). So, here we first analyzed the effects of CBD directly on the PPI response of capuchin monkeys and then evaluated the influence of a CBD pre-treatment on repeated MK-801-induced changes in the same PPI test.

## Materials and Methods

### Ethics Statement

All the procedures herein complied with the Brazilian regulations for the scientific use of laboratory animals (Lei Arouca 11.794/2008), as well as the CONCEA/Brazil and NIH/USA guidelines for the care and use of laboratory animals, and were approved by the Animal Ethics Committee of the University of Brasilia (no. 131791/2013).

### Subjects and General Housing Conditions

In total, seven capuchin monkeys (*Sapajus* spp.) were used, one male and six females, weighing between 2.5 and 5.0 kg at the beginning of the study. All subjects were housed and tested at the Primate Center of the University of Brasilia, Brazil. They were group or paired-housed under natural light, temperature and humidity conditions in standard cages (3 m × 3 m × 1.8 m) containing rope swings, nest boxes and natural substrate on the floor. Fresh food was provided daily at 07:00 h, consisting of a mixture of pieces of fruits and vegetables. Boiled eggs, nuts and cooked chicken breast were given several times a week, also at 07:00 h. Unconsumed items were removed at 17:00 h, while water and chow were available *ad libitum*.

Housing and maintenance conditions complied with the regulations of the Brazilian National Institute of Environment and Renewable Natural Resources (IBAMA). The animals were only deprived of food and water during the specific trials indicated in the procedure below. All subjects used had previous experience with the PPI protocol (Saletti et al., 2014).

### Startle Measurement and General Procedure

Prepulse inhibition testing was performed in a transparent acrylic chamber (60 cm × 30 cm × 30 cm) placed above a wooden box (45 cm × 40 cm × 40 cm). Each subject was placed inside this chamber in such a way that its head protruded through an adjustable central neck hole located at the top. A transparent acrylic device (30 cm × 30 cm × 25 cm) was placed directly on top of the chamber, encompassing the subject’s head. This device contained three speakers (model FT96H- frequency band 4 KHz∼30 KHz; Fostex, Japan), each located 10 cm from the monkey’s head and connected to a sound generator (O’Hara & Co., Ltd., Japan). One speaker was positioned on each side of the animal’s head and generated the startle stimuli. The third speaker was placed at the back of the device and emitted a constant 65dB white noise. An accelerometer (model BDK3; Inntechno Japan Co.Ltd., Japan), connected to an amplifier (O’Hara & Co., Ltd., Japan), was placed at the bottom of the chamber. When the subject was inside the chamber it stood on the accelerometer platform. The accelerometer captured the animal’s whole-body movement and transmitted the data to be recorded on the Animal Startle software (PCI 6024E, developed by O’Hara & Co., Ltd., Japan) that interfaced with the Windows XP system (for details see Saletti et al., 2014). A video camera (model #1004124; Clone, Brazil) was connected to this experimental setup and monitored the animal during each session.

Testing was conducted 5 days a week, between 8:00 and 12:00 h, in an acoustically isolated room located near the monkeys’ home-cage. After a 10 min acclimatization period to the test room and setup described above, PPI testing was conducted. It consisted of 10 consecutive blocks of stimuli presentation, held at 60 s intervals. During each block, three different stimuli were randomly presented, also at 60 s intervals: a 115 dB pulse of 40 ms duration, an 80 dB prepulse of 20 ms duration and a prepulse-pulse combination with a 120 ms interval between the prepulse and pulse presentation. Stimuli intensity and duration were based on previous studies (Saletti et al., 2014, 2015). The startle response was recorded by the Animal Startle software system that measured the animals’ body movements through changes in force detected by the accelerometer. The animals’ body movements were recorded during a 600 ms post-stimuli interval and the peak amplitude registered following the pulse or prepulse+pulse stimulus of each trial was recorded as the startle amplitude.

### Experiment 1: Effects of CBD Treatment on PPI

In the first experiment, CBD (0, 15, 30, and 60 mg/kg; volume of 1 mL/kg; STI Pharm, UK) was dissolved in a 1:19 solution of Tween 80 (Sigma-Aldrich, Brazil) and 0.9% saline, respectively. Doses were based on previous studies in rodents (Moreira and Guimarães, 2005; Almeida et al., 2013; Levin et al., 2014). For the safety of the personnel involved and to insure correct intraperitoneal (ip) administrations, all animals were briefly exposed to the anesthetic isoflurane via inhalation, yet an effective anesthesia stage was never really attained.

All subjects were initially tested with vehicle and then treated with the three doses of CBD. Only one treatment was given on each test day, with a 2-week interval between trials. The order in which the three CBD doses were given was randomly assigned for each animal. Each subject received the vehicle injection or a CDB dose and was then placed in the test chamber. After a 30 min interval, it was submitted to the PPI test procedure described above. Two females were excluded from the analyses due to data recording problems, totalizing five animals in experiment 1 (one male and four females).

### Experiment 2: Effects of CBD Pre-treatment Following Repeated MK-801 Treatment

In this second experiment, the same seven monkeys were tested 4 months after the previous study. MK-801 (0.02 mg/kg; Sigma-Aldrich, Brazil) and CBD (60 mg/kg; STI Pharm, UK) were both dissolved in a 1:19 solution of Tween 80 (Sigma-Aldrich, Brazil) and 0.9% saline, respectively. The former was administered intramuscularly (im), whereas CBD was again given via ip route. The injection volume of both substances was 1 ml/kg. The MK-801 dose was based on our previous study (Saletti et al., 2015).

Each animal was given a MK-801 injection, once a week, during three consecutive weeks. Twenty minutes after each injection, the subject was tested in the same PPI procedure described above. On the 4th week of testing, each monkey was pre-treated with CBD and then 10 min later it received a MK-801 treatment. PPI testing was once again held 20 min after the MK-801 administration. Two weeks later, each animal was injected with vehicle and following a 20 min interval tested in the PPI protocol (VEH 2). To control for a habituation effect due to repeated testing, and considering that the same monkeys were used in both experiments, data from the vehicle trial of Experiment 1 were used as a second control session in this study (VEH 1).

### Behavioral and Statistical Analysis

Data were expressed as the mean startle response or percentage of PPI + standard error of the mean (+S.E.M.). The data were normalized using the following calculation of the percentage of inhibition (Saletti et al., 2014, 2015):

$$\frac{100*\left(p-pp\right)}{p}$$

where *p* corresponds to the pulse-alone startle response and *pp* to the prepulse+pulse response.

The data were normally distributed, according to the Shapiro–Wilk test. To establish a possible between-treatment effect (vehicle, MK-801 and CBD+MK-801) on the capuchins’ startle amplitude and percentage of PPI, data from each experiment were analyzed using a one-way ANOVA for repeated measures (pulse and prepulse+pulse trials were analyzed separately). Whenever significant results were obtained, Fisher’s LSD *post hoc* test was used for pair-wise comparison across treatments. Significance level for all tests was set at *p* < 0.05. For Shapiro–Wilk and ANOVA analysis we used the IBM SPSS^®^ version 20 software. For comparisons with *p* < 0.05, we also calculated the effect sizes (effsize) using Matlab software^®^ (version R2013a).

## Results

### Experiment 1: Effects of CBD Treatment on PPI

**FIGURE 1:**
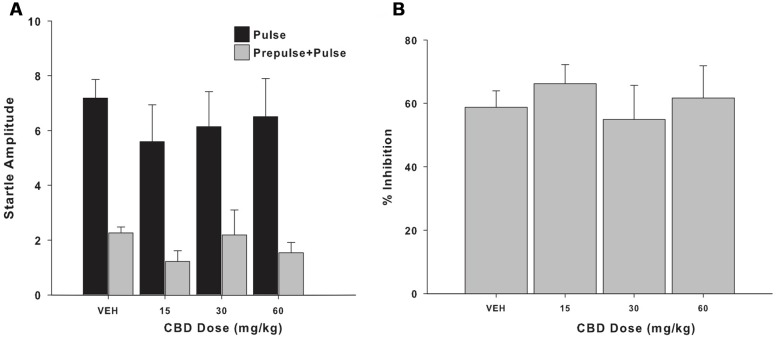
**(A)** Mean (+SEM) startle amplitude of the capuchin monkeys (*n* = 5) in the pulse (black bars) and prepulse-pulse trials (gray bars) following the vehicle (VEH) and the three CBD treatments (15, 30, and 60 mg/kg). **(B)** Mean (+SEM) percentage of prepulse inhibition (PPI) of the capuchin monkeys (*n* = 5) following the vehicle (VEH) and the three CBD treatments (15, 30, and 60 mg/kg).

**FIGURE 2:**
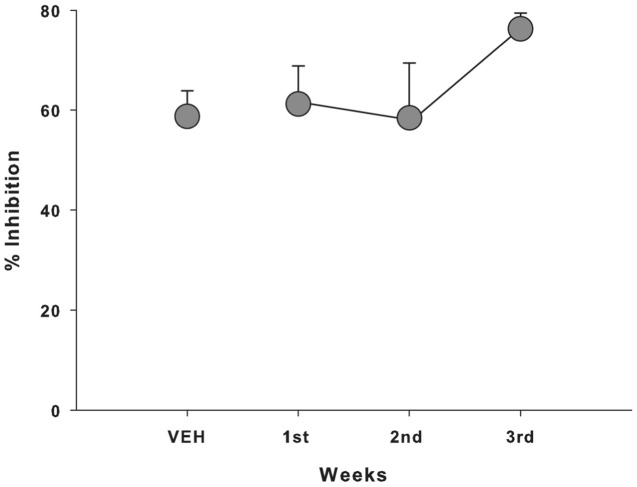
**Mean (±SEM) percentage of PPI of the capuchin monkeys (*n* = 5) according to the week of Experiment 1, regardless of the CBD dose received; VEH, vehicle**.

In terms of the startle amplitude, a significant between-treatment effect was not observed for either the pulse (*F*_3,12_ = 0.566, *p* = 0.648; **Figure 1A**) or prepulse+pulse trials (*F*_3,12_ = 1,375; *p* = 0.297; **Figure 1A**), with the subjects’ response remaining constant regardless of the treatment received. A similar profile was seen for the percentage of PPI when analyzing the data according to the treatment received (*F*_3,12_ = 0.368, *p* = 0.778; **Figure 1B**) and over the course of the weeks of the experiment, regardless of the specific treatment received (*F*_3,12_ = 1.283, *p* = 0.325; **Figure 2**).

### Experiment 2: Effects of CBD Pre-treatment on MK-801-Induced Changes in PPI

**FIGURE 3:**
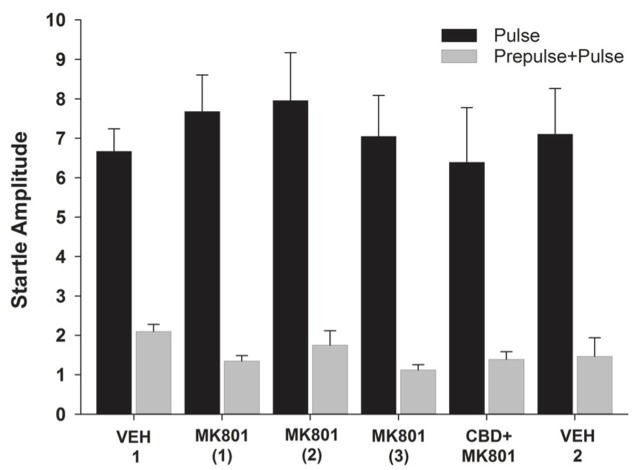
**Mean (+SEM) startle amplitude of the capuchin monkeys (*n* = 7) in the pulse (black bars) and prepulse-pulse trials (gray bars) after vehicle administration (VEH 1), the injections of MK-801 (1–3) held once a week during three consecutive weeks, the CDB pre-treatment followed by a MK-801 treatment (CBD+MK801) held on the 4th week, and the second vehicle administration (VEH 2) performed on the 6th week.** Except for VEH 1, held during Experiment 1, all other data were recorded during Experiment 2.

**FIGURE 4:**
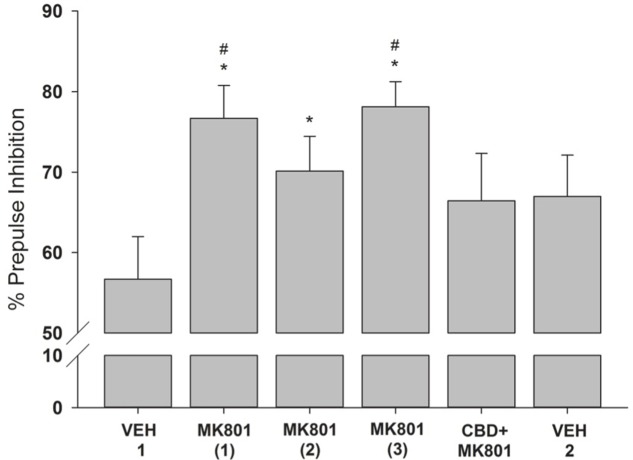
**Mean (+SEM) percentage of PPI of the capuchin monkeys (*n* = 7) following the vehicle administration (VEH 1), the injections of MK-801 (1–3) held once a week during three consecutive weeks, the CDB pre-treatment followed by a MK-801 treatment (CBD+MK801) held on the 4th week, and the second vehicle administration (VEH 2) performed on the 6th week.** Except for VEH 1, held during Experiment 1, all other data were obtained during Experiment 2. ^∗^*p* < 0.05 vs. VEH 1; #*p* < 0.05 vs. CBD+MK-801.

Among the different pulse (*F*_5,30_ = 1.270, *p* = 0.303; **Figure 3**) or prepulse+pulse trials (*F*_5,30_ = 1.800, *p* = 0.143; **Figure 3**), significant differences were not observed. However, the percentage of PPI differed significantly between treatments (*F*_5,30_ = 4.052, *p* = 0.006; **Figure 4**). This parameter was significantly higher following all three MK-801 administrations, compared to the first vehicle administration [vs. MK-801(1): *p* = 0.009, effsize = 1.498; vs. MK-801(2): *p* = 0.026, effsize = 0.983; vs. MK-801(3): *p* = 0.005, effsize = 1.743]. The percentage of PPI after the CDB+MK-801 treatment was significantly lower than on the trials with only MK-801(1) and MK-801(3), being similar to the levels seen following both vehicle injections [vs. VEH 1: *p* = 0.183; vs. MK-801(1): *p* = 0.02, effsize = 0.718; vs. MK-801(2): *p* = 0.512; vs. MK-801(3): *p* = 0.013, effsize = 0.878; vs. VEH 2: *p* = 0.954].

## Discussion

### CBD on Monkey PPI

The results from Experiment 1 indicated that the CBD administration alone had no effect on the capuchin monkeys’ startle amplitude or PPI response. Although to our knowledge this is the first study to evaluate such aspect in NHPs, similar results have been observed in rodents (Long et al., 2006; Gomes et al., 2014; Levin et al., 2014; Pedrazzi et al., 2015). Long et al. (2006) and Gururajan et al. (2011) reported, however, that a systemic administration of CBD altered the startle response of rodents. In this case, the percentage of PPI decreased following a 10 mg/kg dose of CBD, whereas 3 and 30 mg/kg had no effect.

This discrepancy may be partly due to species-specific differences, as the CB1 receptor density of rodents and primates differs in certain regions of the brain (Ong and Mackie, 1999), as well as their motor response to cannabinoid-related substances (Meschler et al., 2001). Important methodological aspects may also have contributed. Due to ethical restrictions and reduced sample size in monkey research, our subjects were submitted to the PPI procedure in a repeated-exposure design. Nonetheless, a temporal or drug-training effect does not seem to have influenced the present result as the percentage of PPI remained constant over the course of the procedure, regardless of the specific CBD treatment given. Thus, our results in capuchins suggest that CB1 receptor antagonism may have no effect on sensorimotor gating mechanisms.

### CBD Pre-treatment on MK-801 Induced PPI Enhancement

MK-801 has been shown to induce schizophrenic-like behaviors in monkeys, such as deficits in memory-related processing (Ogura and Aigner, 1993; Buffalo et al., 1994; Harder et al., 1998; Harder and Ridley, 2000; Tsukada et al., 2005; Wang et al., 2012) and more recently in changes in PPI responding (Saletti et al., 2015). Although an acute 0.03 mg/kg dose of MK-801 decreased the PPI of capuchin monkeys, repeated testing (with different doses) in the same setup diminished this effect possibly due to drug-induced ataxia or tolerance (Saletti et al., 2015).

In the present study, we opted to use a lower dose of MK-801 to reduce such levels of ataxia. The 0.02 mg/kg dose was used in a repeated administration regimen, thereby leading to an increase in the monkeys’ percentage of PPI. This result differs from the MK-801-induced decrease in PPI reported in rodents (Schulz et al., 2001; Gomes et al., 2014), considered to be an indicator of a psychotic-like effect of this drug. It also contrasts with the lack of effect observed for this dose in our previous study using the same animals (Saletti et al., 2015). In that instance, each subject received all possible treatments only once (vehicle, 0.01, 0.02, and 0.03 mg/kg) in a pseudo-randomized order, whereas only the 0.02 mg/kg dose was presently given on three consecutive trials. Thus the present increase in PPI, detected already on the first trial, seems unlikely to be due to a habituation effect considering the 5-month interval between the experiments, the similar PPI and startle amplitude levels seen during the vehicle control trials of the two studies (≈55% and 6 points, respectively), and the response stability seen on the last session of current study (VEH 2 vs. CBD+MK801). Ketamine, another NMDA antagonist, is also reported to increase the PPI response of healthy human volunteers when using a brief inter-stimulus interval (Braff et al., 2001; Duncan et al., 2001). The use of higher doses in rodents and distinct hippocampal NMDA receptor sensitivity could be important aspects contributing to the discrepancies between human and rodent PPI responding (Duncan et al., 2001). NMDA antagonists can block hippocampal long-term potentiation, with this effect being modified by pre-training or test familiarization through latent learning effect (Otnaess et al., 1999; Ennaceur et al., 2011). The hippocampus is also known to be responsable for PPI response modulation (Kohl et al., 2013).

Interestingly, pre-treatment with CBD prevented the MK-801-induced increase in the monkeys’ PPI. Considering the large effect sizes values found (≈0.8 or more; Sullivan and Feinn, 2012), we are able to conclude that the difference between the treatments is expressive. Nonetheless, the effects induced by both MK-801 and CBD seem related to their anxiogenic and anxiolytic profiles, respectively – an aspect that is highly relevant in the context of a startle response. In mice, MK-801 is reported to increase anxiety levels (Ennaceur et al., 2011), whereas CBD has been frequently linked to anxiolysis (Zuardi et al., 1982; Guimarães et al., 1990; Campos and Guimarães, 2008; Almeida et al., 2013). CBD seems to activate serotoninergic 5-HT1A receptors (Russo et al., 2005; Resstel et al., 2009; Soares et al., 2010; Gomes et al., 2011) and attenuate cardiovascular and/or behavioral responses associated with anxiety and panic in rats (Resstel et al., 2009; Soares et al., 2010).

Although CBD has been reported to reverse the PPI disruption induced by MK-801 (Long et al., 2006; Gomes et al., 2014) and amphetamine in rodents (Pedrazzi et al., 2015), our present results with capuchins fail to corroborate a possible anti-psychotic effect for this compound in the context of sensorimotor gating. In the presence of only MK-801, the startle amplitude generally tended to decrease in the ‘pulse’ trials, yet increase in ‘prepulse+pulse’ trials. The latter, however, was also observed with subsequent treatments (CBD+MK-801 and VEH 2). The pre-treatment of the monkeys with CBD reversed this increase tendency in startle amplitude in PPI, with no visible changes in ‘pulse’ trials. Altogether, these results suggest that the CBD effects on PPI are unlikely to stem from a direct involvement in sensorimotor gating mechanisms. We suggest that the CBD-induced reversal of the MK-801 effects may be related to their anxiolytic and anxiogenic profiles, respectively (Zuardi et al., 1982; Ennaceur et al., 2011; Almeida et al., 2013), although this aspect was not directly investigated in the present study. Further studies are necessary to better elucidate the interplay between anxiety and schizophrenia, as well as the potential use of CBD as an antipsychotic drug, possibly using other NHP models as well.

## Conclusion

In the present study, CBD alone had no effect on the PPI response of capuchin monkeys, yet blocked the increase in this response that was induced by NMDA receptor antagonism (MK-801). This effect may stem from a general anxiolytic rather than an anti-psychotic effect, corroborating an anxiolytic profile of CBD in the PPI paradigm.

## Author Contributions

CT, PS, and RM: Conception and design, acquisition of data, or analysis and interpretation of data; drafting the article or revising it critically for important intellectual content. MB and HN: Drafting the article or revising it critically for important intellectual content.

## Conflict of Interest Statement

The authors declare that the research was conducted in the absence of any commercial or financial relationships that could be construed as a potential conflict of interest.
